# Genetic Diversity and Characterization of Symbiotic Bacteria Isolated from Endemic *Phaseolus* Cultivars Located in Contrasting Agroecosystems in Venezuela

**DOI:** 10.1264/jsme2.ME20157

**Published:** 2021-06-05

**Authors:** María Daniela Artigas Ramírez, Mingrelia España, Hitoshi Sekimoto, Shin Okazaki, Tadashi Yokoyama, Naoko Ohkama-Ohtsu

**Affiliations:** 1 Iriomote Station, Tropical Biosphere Research Center, University of the Ryukyus, 870 Uehara, Yaeyama, Taketomi, Okinawa, 907–1541, Japan; 2 Institute for Advanced Studies (IDEA), Miranda—Venezuela; 3 Faculty of Agriculture, Utsunomiya University, Utsunomiya 321–8505, Japan; 4 Institute of Agriculture, Tokyo University of Agriculture and Technology (TUAT), Saiwai-cho 3–5–8, Fuchu, Tokyo, 183–8538, Japan; 5 Faculty of Food and Agricultural Science, Fukushima University, Kanayagawa 1, Fukushima city, Fukushima, 960–1296, Japan; 6 Institute of Global Innovation Research and Institute of Agriculture, Tokyo University of Agriculture and Technology (TUAT), Saiwai-cho 3–5–8, Fuchu, Tokyo, 183–8538, Japan

**Keywords:** *Ensifer*, *Phaseolus*, *Burkholderia*, *Mesorhizobium*, *Bradyrhizobium*, Venezuela

## Abstract

*Phaseolus vulgaris* is a grain cultivated in vast areas of different countries. It is an excellent alternative to the other legumes in the Venezuelan diet and is of great agronomic interest due to its resistance to soil acidity, drought, and high temperatures. *Phaseolus* establishes symbiosis primarily with *Rhizobium* and *Ensifer* species in most countries, and this rhizobia-legume interaction has been studied in Asia, Africa, and the Americas. However, there is currently no evidence to show that rhizobia nodulate the endemic cultivars of *P. vulgaris* in Venezuela. Therefore, we herein investigated the phylogenetic diversity of plant growth-promoting and N_2_-fixing nodulating bacteria isolated from the root nodules of *P. vulgaris* cultivars in a different agroecosystem in Venezuela. In comparisons with other countries, higher diversity was found in isolates from *P. vulgaris* nodules, ranging from *α-* and *β-proteobacteria*. Some isolates belonging to several new phylogenetic lineages within *Bradyrhizobium*, *Ensifer*, and *Mesorhizobium* species were also specifically isolated at some topographical regions. Additionally, some isolates exhibited tolerance to high temperature, acidity, alkaline pH, salinity stress, and high Al levels; some of these characteristics may be related to the origin of the isolates. Some isolates showed high tolerance to Al toxicity as well as strong plant growth-promoting and antifungal activities, thereby providing a promising agricultural resource for inoculating crops.

*Phaseolus vulgaris* (common bean) is indigenous to America and is currently the most cultivated legume worldwide after soybean (Velázquez *et al.*, 2005). There are approximately 1,300 wild types of *P. vulgaris*. The remaining types are distant relatives of the common bean (CIAT, 2001). Approximately 50 wild-growing species are distributed across American countries (Cobley and Steele, 1976). This genus includes between 150 and 200 cultivated species used as food or garden ornamentals. *Phaseolus* species have been domesticated in different ecosystems ranging from mesic and temperate to warm and cold, humid, or hot and dry and exhibit distinct adaptations and reproductive systems. *Phaseolus* represents a wide range of habits, including annual and perennial, and some beans (*e.g.*, large lima) may behave as short-lived perennials, bushes, or climbers (Cobley and Steele, 1976; Miklas and Singh, 2007). Several species are important for human or animal consumption, such as common beans (*Phaseolus vulgaris* L.), lima beans (*P. lunatus* L.), runner beans (*P. coccineus* L.), tepary beans (*P. acutifolius* A. Gray), and year-long beans (*P. polyanthus* Greenman) (Debouck *et al.*, 1993). *Phaseolus* is primarily used as a food crop throughout Latin American and African countries. It is considered the core of bean diversity from which wild beans dispersed northwards and southwards to form the two geographically distinct gene pools of Meso­america and Andean South America, including the Venezuelan Andes (Gepts, 1998). However, *P. vulgaris* is a warm-season crop that cannot tolerate frost or extensive exposure to near-freezing or overheating temperatures at any growth stage (CIAT, 2001). Additionally, low P and N availability constrain common bean production, mainly in Hispanic America and Africa (Wortmann and Allen, 1994; Patel *et al.*, 2010). *Phaseolus* is widely cultivated in Venezuela and mainly grown on erosion-prone slopes or in places with limited fertility. In the highlands, the plants are nodulated by native rhizobial populations without anthropological interventions. However, this diversity remains uninvestigated.

Nodulating bacteria associated with *Phaseolus* species have generally been classified as *Rhizobium*, *Bradyrhizobium*, *Ensifer*, *Paraburkholderia*, and *Pararhizobium* (Talbi *et al.*, 2010; Verástegui-Valdés *et al.*, 2014; Mhamdi *et al.*, 2015; Andrews and Andrews, 2017). However, this diversity of genus or species has not been found in the same country at the same time. *P. vulgaris* establishes symbiosis with more N_2_-fixing and fast-growing *Rhizobium* and *Ensifer* genera (Verástegui-Valdés *et al.*, 2014), including a wide range of nodulating bacteria, than other beans. Researchers in other countries reported three *Rhizobium* species: *R. etli*, *R. phaseoli*, and *R. tropici* as predominant *Phaseolus* symbionts (Martínez-Romero *et al.*, 1991; Ribeiro *et al.*, 2013). However, recent evidence indicates that other species formerly classified as *Agrobacterium* are capable of nodulating leguminous plants, such as *R. radiobacter* (formerly *Agrobacterium tumefaciens*), which nodulates *P. vulgaris*, *Campylotropis* spp., *Cassia* spp. (Han *et al.*, 2005; Verástegui-Valdés *et al.*, 2014), and *Wisteria sinensis* (Liu *et al.*, 2005). Another species, *R. rhizogenes* strains containing a Sym plasmid, also form nodules on *P. vulgaris* (Velázquez *et al.*, 2005).

Rhizobia associated with several legumes have been reported in Venezuela (Vinuesa *et al.*, 2005; Marquina *et al.*, 2011; Artigas *et al.*, 2020). Marquina *et al.* (2011) described rhizobia associated with one variety of *P. vulgaris* from Inceptisol. Venezuelan *Glycine max* and *Vigna* rhizobia were derived from different genera, including novel strain lines within the *Burkholderia*/*Paraburkholderia* group (Artigas *et al.*, 2019). However, limited information is currently available on the nodulating bacteria related to endemic legumes in Venezuela, such as *Phaseolus* spp. Therefore, we herein examined the phylogenetic diversity and physiological characteristics of *Phaseolus* rhizobia from different Venezuelan ecosystems and topographical regions. The present study focused on two main crop cultivars continuously cultivated in different regions. We also investigated the relationship between the phylogeny and distribution of rhizobia in Venezuela and host specificity between two endemic *Phaseolus* varieties.

## Materials and Methods

### Soil samples and collection sites

**Fig. 1.**
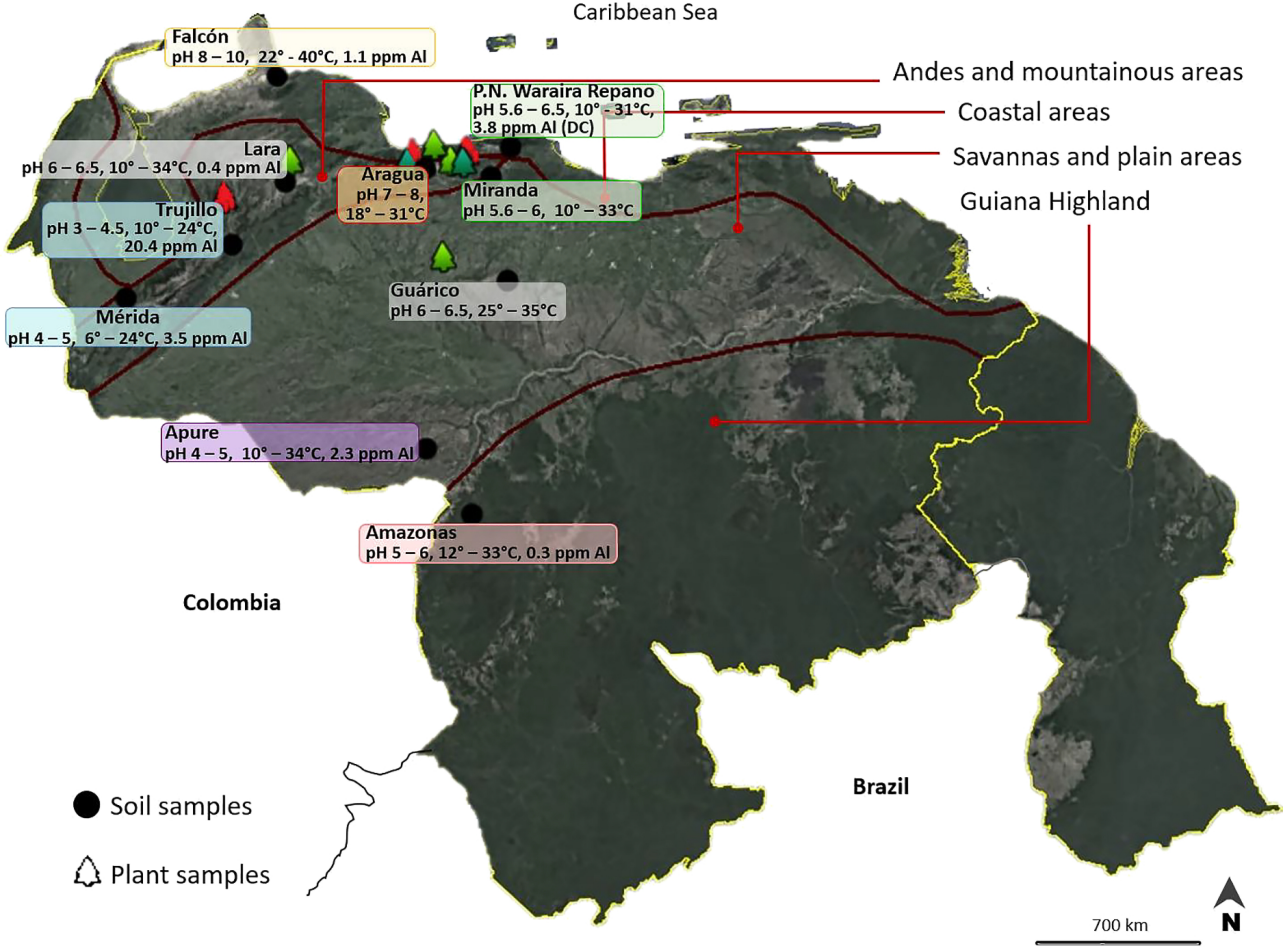
Map of Venezuela showing different agro-ecological regions and geographical locations for sites at which soil and nodules were sampled. This map was modified from a previous figure reported by Artigas *et al.* (2020) using Google Earth software ver. Pro. Red plants indicate the areas at which *Phaseolus* (Black cultivar) are continuously cultivated. pH and Al values were reported by Casanova (2005), and regions without Al values indicate that Al was not detected from soils in these areas. REDBC and INIA-Venezuela reported average temperatures.

Root nodules were collected from *P. vulgaris* ‘Tacarigua’ (Black cultivar) and *P. vulgaris* ‘L2234-MGM’ (White cultivar) in Aragura and Lara and from the White cultivar in Guárico (Table 1 and Fig. 1).

Soil samples were collected from ten Venezuelan regions (Table 1) and used in another study by Artigas *et al.* (2019; 2020). No inoculants were used in these soil-collected areas; therefore, the strains obtained were considered to be indigenous to Venezuela. The ecosystems of the soil-collected areas and soil types are shown in Fig. 1 and Table 1. Al and pH values in Fig. 1 are reported in Casanova (2005), which were assessed using standard Al/pH-soil methods (Hsu, 1963; Jones, 2001).

### Isolation of rhizobia from Venezuelan soils using endemic *Phaseolus* cultivars as trap hosts

*P. vulgaris* ‘Tacarigua’ (Black cultivar) and ‘L2234-MGM’ (White cultivar) are used as trap hosts to isolate rhizobia strains. Seeds were surface-sterilized with 70% (v/v) ethanol for 1‍ ‍min and 3% (v/v) sodium hypochlorite for 2‍ ‍min, followed by washing four times with sterile distilled water (Artigas *et al.*, 2019). The seeds were pre-germinated in darkness under sterile conditions at 28°C for 48 h on filter paper (Whatman^TM^ No.GF/A, glass microfiber filter, 90‍ ‍mm) moistened with 5‍ ‍mL of sterile Milli-Q water (the Direct-Q^®^ 3 UV system; Merck Millipore) in a sterile Petri dish. Filter paper and water were sterilized with an autoclave at 120°C and 0.2 MPa for 30‍ ‍min (Sylvester–Bradley *et al.*, 1983) before use. Pre-germinated plants were inoculated with 2‍ ‍g of each soil suspended in 10‍ ‍mL of sterile water and then transferred into 300-‍mL glass jars containing 120‍ ‍g of sterilized vermiculite (Vermitech). Sterilized N-free nutrient solution (Somasegaran and Hoben, 1994) was added to the jar at 0.6‍ ‍mL g^–1^ vermiculite, and this moisture level was maintained throughout the growth period by supplementing with N-free solution. Plants were grown at 28°C for four weeks in a growth room with a 16-h light (5000~7000 LUX)/8-h dark photoperiod. After four weeks, root nodules were harvested and their surface was sterilized with 70% (v/v) ethanol for 30‍ ‍s, washed with sterile distilled water, and then immersed for 1‍ ‍min in 5% (v/v) sodium hypochlorite, followed by washing four times with sterile distilled water. Surface-sterilized root nodules were crushed in 500‍ ‍μL of glycerol solution (50% [v/v]) to obtain bacterial suspensions. An aliquot (10‍ ‍μL) of each suspension was streaked onto Yeast Mannitol agar (YMA) (Vincent, 1970) and incubated for one week at 28°C. The remaining suspension was frozen at –80°C for further isolation (if necessary). Single colonies were re-streaked onto fresh plates to obtain pure colonies. Strains were phenotypically characterized for their growth rate, texture, and color on YMA plates. All isolates were re-inoculated onto the hosts following the authentication protocol using the modified plant pot experiment type (CIAT, 1988).

### Authentication of symbiotic activity and performance

*P. vulgaris* ‘Tacarigua’ (Black cultivar) was used to examine the symbiotic activity and performance of isolated strains. One hundred and twenty isolates were selected (Table 1) and grown in YM broth at 28°C for 5 days to obtain 10^9^‍ ‍cells‍ ‍mL^–1^, as described by Vincent (1970). Cells were collected by centrifugation at 10,000‍ ‍rpm at 4°C for 5‍ ‍min, followed by resuspension in TE buffer (1‍ ‍mM EDTA in 10‍ ‍mM Tris-HCl [pH 8.0]). Prior to being inoculated, *Phaseolus* seeds were surface-sterilized with 70% (v/v) ethanol for 30‍ ‍s and 3% (v/v) sodium hypochlorite for 2‍ ‍min followed by washing four times with sterile distilled water. One milliliter of each rhizobial cell suspension containing 10^8^ cells in TE buffer was then inoculated on each seed of ‘Black’ *P. vulgaris*. Inoculated seeds were sown in plant boxes (7.6×7.6×10.2‍ ‍cm) with 200‍ ‍g of vermiculite (Vermitech). Sterilized N-free nutrient solution (Somasegaran and Hoben, 1994) was added at 0.6‍ ‍mL g^–1^ vermiculite, and moisture was maintained throughout the culture by supplementing with N-free solution. One plant in each plant box was grown at 28°C in a growth chamber (Fli 2000; EYELA, Tokyo Rikakikai) with a 16-h light/8-h dark photoperiod. Plants with no inoculation served as the treatment control (Vincent, 1970).

The entire plant with the root nodules was collected 30 days after the inoculation to assess N_2_ fixation activity based on acetylene reduction activity (ARA). ARA was detected using a Shimadzu GC-2014 gas chromatograph (Shimadzu) equipped with a Porapak N column (Agilent Technologies) with a 30-min incubation (Artigas *et al.*, 2020). Root nodule numbers were confirmed. Shoot and root weights were measured after they had been dried at 80°C for 48 h.

### Isolation of genomic DNA

Sixty-three isolates were selected based on their nodulation ability. DNA was extracted from isolates grown in YM broth medium at 28°C for 4 days. Before genomic isolation, cells were collected and washed twice with equal volumes of PBS buffer (NaCl 137‍ ‍mM, KCl 2.7‍ ‍mM, Na_2_HPO_4_ 10‍ ‍mM, and KH_2_PO_4_ 1.8‍ ‍mM [pH 7.2]). Total genomic DNA was extracted from isolates using the CTAB method described by Artigas *et al.* (2019), and DNA concentrations and purities were confirmed using a NanoDrop 2000 UV–vis spectrophotometer (Thermo Fisher Scientific).

### DNA amplification and sequencing

Primer sets for the 16S rRNA gene are described in Young *et al.* (1991) and Artigas *et al.* (2019), and primers for the genes of DNA recombinase A (*rec*A), ATP synthase (*atp*D), and glutamate synthase (*gln*A) are described in Gaunt *et al.* (2001) for *α-proteobacteria* and Baldwin *et al.* (2005) for *β-proteobacteria*. The *nif*H primer set described in Laguerre *et al.* (2001) and the *nod*D gene primer set described in Risal *et al.* (2012) and Zézé *et al.* (2001) were used. Amplification was performed using the thermal cycler (GeneAmp PCR system 9700; Applied Biosystems) described by Artigas *et al.* (2020). PCR products were examined using a 1.5% (w/v) agarose gel with 0.5× TBE buffer (10×: 1 M boric acid, 0.02 M EDTA·2Na, and 1 M Tris-HCl base [pH 8.0]) mixed with 0.5‍ ‍μg mL^–1^ ethidium bromide. Bands with the predicted sizes were then excised from gels, and DNAs were purified using a FastGene^®^ agarose gel/PCR extraction kit (Nippon Genetics). According to the manufacturer’s protocols, PCR products were sequenced using the ABI Prism 3500 Genetic Analyzer (Applied Biosystems). The sequences obtained were aligned using the ClustalW method and then compared in the GenBank database (https://www.ncbi.nlm.nih.gov/genbank/) using the online software BLAST algorithm-based sequence alignment. Phylogenetic trees were constructed by Genetyx version 11 and MEGA version 12.0 (Tamura *et al.*, 2013) based on a neighbor-joining analysis and using the bootstrap method with the Maximum Composite Likelihood model without topology. Multilocus sequence typing (MLST) was conducted based on 16S rRNA and housekeeping genes (Artigas *et al.*, 2019).

Accession Numbers: The sequences obtained for the different genes found in the present study have been deposited in the DNA Databank of Japan (DDBJ) under the following accession numbers: LC585433–LC585495 for 16S rRNA sequences, LC585496–LC585558 for the *atp*D gene, LC585559–LC585621 for the *rec*A gene, LC585622–LC585684 for the *gln*A gene, LC585685–LC585747 for the *nif*H gene, and LC585748–LC585810 for the *nod*D gene.

### Abiotic stress tolerance profiles of *Phaseolus* rhizobia

Isolates were initially grown in YM broth at 28°C for 5 days, and 5‍ ‍μL of cell suspensions at 10^8^ cells L^–1^ was transferred onto YMA plates or broth followed by an incubation at 28°C for 5–10 days with different stress conditions, such as high temperature, alkalinity, acidic pH, high salinity, and a high concentration of Al at different pH levels, as described in Artigas *et al.* (2019). The temperature tolerance of isolates was based on their ability to grow under the following temperatures: 25, 28, 35, 40, and 45°C on YMA plates, with 28°C being set as the control. The ability to grow at different pH levels was examined at pH 4.5, 5, 6.8, 8, 9, or 10, with pH 6.8 being set as the control (Somasegaran and Hoben, 1994), and pH in YMA plates was adjusted with 0.5‍ ‍M HCl or 0.5‍ ‍M NaOH. In the salinity tolerance test, YMA was supplemented with NaCl at 0 (control), 1, 2, 3, or 4% (w/v). Al tolerance was evaluated with 0 (control), 0.1, 0.5, 1, or 2‍ ‍mM of AlCl_3_·6H_2_O (Wako Pure Chemical) under acidic (pH 4.5) or neutral (pH 6.8) conditions. After 5 days, colony-forming units (CFUs) were calculated by plate counting under stress conditions. The growth of isolates was estimated relative to the control treatment (non-stress) as follows: no growth; weak growth (10–20% of the control); good growth (30–60% of the control); and excellent growth (similar to/the same as the control) (Somasegaran and Hoben, 1994; Marquina *et al.*, 2011). These experiments were performed in triplicate for each isolate.

### Antibiotic tolerance profiles of *Phaseolus* rhizobia

The antibiotic resistance or sensitivity of isolates was evaluated by testing their ability to grow under the following concentrations of different antibiotics: kanamycin sulfate (Kan, 30‍ ‍μg mL^–1^; Fujifilm Wako Pure Chemical), spectinomycin (Spe, 40‍ ‍μg mL^–1^; Sigma-Aldrich), streptomycin (Str, 40‍ ‍μg mL^–1^, Fujifilm Wako Pure Chemical), chloramphenicol (Cp, 80‍ ‍μg mL^–1^, Fujifilm Wako Pure Chemical), and nalidixic acid (Nal, 30‍ ‍μg mL^–1^; Sigma-Aldrich) (Yokoyama *et al.*, 1999). Resistant strains (R) showed a weaker or better growth rate than the control, while sensitive strains (S) did not grow. These experiments were performed in triplicate for each isolate.

### Indole-3-acetic acid (IAA) production

Each strain was inoculated into YM broth containing 100‍ ‍mg L^–1^ L-tryptophan and incubated at 28°C for 5 days in darkness. Cell suspensions were then centrifuged at 10,000‍ ‍rpm for 15‍ ‍min, and IAA concentrations in the supernatants were measured using the Salkovski colorimetric technique (Glickmann and Dessaux, 1995) by measuring absorbance at 530‍ ‍nm with a spectrophotometer (Ultrospec 3300 pro; Amersham Biosciences). These experiments were performed in triplicate for each strain.

### Antifungal profile of *Phaseoli* isolates

Bacterial isolates were grown in YM broth medium at 28°C for 5 days. Each cell suspension at 10^7^‍ ‍cells‍ ‍mL^–1^ was applied to test antifungal activity using the Kirby-Bauer disk diffusion susceptibility modified test protocol described in Hudzicki (2009) with Potato dextrose agar (PDA) and YMA plates. Different pathogen types were obtained from the National Institute of Agrobiological Sciences Genebank from Tsukuba, Japan (stock by MAFF, Japan), such as *Pythium aphanidermatum* (MAFF No. 239200), *Rhizoctonia solani* (MAFF No. 237699), *Fusarium graminearum* (MAFF No. 240353), *Pyricularia oryzae* (MAFF No. 101506), *Colletotrichum gloeosporioides* (MAFF No. 306534), *Rosellinia necatrix* (MAFF No. 328101), and *Helicobasidium mompa* (MAFF No. 328090). Two controls were set: a plate with an isolate without a pathogen and a plate with a pathogen without an isolate. The zone inhibition diameter and colony size were measured, and the percent inhibition of the growth of the test pathogen was calculated. The experiment was performed in triplicate for each rhizobia strain and pathogen.

### Phosphorous (P) and potassium (K) solubilization performance

Bacterial isolates were grown in YM broth medium at 28°C for 5 days, and 5‍ ‍μL (10^7^‍ ‍cells‍ ‍mL^–1^) of each culture was then spotted onto Pikovskaya’s medium to test for P solubilization or Aleksandrow’s agar to test for K solubilization. Pikovskaya’s medium contained 10‍ ‍g glucose, 0.5‍ ‍g (NH_4_)_2_SO_4_, 0.2‍ ‍g NaCl, 0.1‍ ‍g MgSO_4_·7H_2_O, 0.2‍ ‍g KCl, 0.002‍ ‍g MnSO_4_·H_2_O, 0.002‍ ‍g FeSO_4_·7H_2_O, and 0.5‍ ‍g L^–1^ yeast extract supplemented with 5.0‍ ‍g L^–1^ of inorganic phosphorus as tricalcium phosphate (Ca_3_[PO_4_]_2_, Wako Pure Chemical) as insoluble P and pH was adjusted to 7.0 (Premono *et al.*, 1996). Aleksandrow’s agar contained 5.0‍ ‍g glucose, 0.5‍ ‍g MgSO_4_·7H_2_O, 0.1‍ ‍g CaCO_3_, 0.005‍ ‍g FeCl_3_, and 2.0‍ ‍g‍ ‍L^–1^ Ca_3_(PO_4_)_2_ supplemented with 2.0‍ ‍g‍ ‍L^–1^ of Mica powder (Wako Pure Chemical) as insoluble K and pH was adjusted to 7.2 (Hu *et al.*, 2006). Isolates were incubated at 28°C for one week. The formation of a clear halo zone around the bacterial colony indicated solubilization activity on Pikovskaya’s medium or Aleksandrow’s agar, and the solubilization index (SI) was calculated as (Halozone diameter+colony diameter [mm]/colony diameter [mm]) (Premono *et al.*, 1996; Hu *et al.*, 2006). Experiments were performed in triplicate from the incubation of each strain.

### Statistical analysis

Dunnett’s test was performed using StatSoft 12.0.

## Results

### Characterization of *Phaseolus* rhizobia isolated from different Venezuelan soils

The ecosystems, soil types, and histories of legume cultivation at the locations at which soils or nodules were sampled are shown in Table 1, and pH, Al concentrations, and temperatures at these sites are shown in Fig. 1. In Aragua, soils and nodules were sampled from fields with and without fertilizer for *Phaseolus* cultivation. Seven out of the ten sampling sites had a cultivation history of *Phaseolus* or other legumes or *Fabaceae* vegetation, such as *Acacia*, *Mimosa*, or *Inga*. These sites included the Andes (Trujillo and Mérida) and Floodplain (Apure) with acidic soils and high concentrations of exchangeable Al (Fig. 1).

A total of 1,212 root nodules were collected from two endemic *P. vulgaris* cultivars: 700 nodules from ‘Tacarigua’ (Black cultivar) and 512 nodules from ‘L2234-MGM’ (White cultivar), with 173 root nodules being collected from *P. vulgaris* grown in the four field locations and 1,039 nodules being harvested from pot cultivations inoculated with the sampled soils (Table 1). The Aragua Valley ecosystem is located in north-central Venezuela; legumes of various genera, such as *Vigna*, *Canajus*, *Phaseolus*, and *Glycine*, were cultivated with or without fertilizer. This sampling site produced a large number of root nodules, which accounted for 24% of all root nodules obtained in the present study. Mérida (Andes-Temperate) showed the second highest nodulation production (17% of the total). 56 root nodules were obtained in the Amazonas (rainforest); this site is located in the Guiana Highlands, in which crop production has traditionally been performed (*e.g.*, cucumber, tomato, and coriander). In Apure (Floodplain), where nutrients such as N and P are deficient, fewer root nodules were obtained than in most of the other sites. In Lara and DC, no nodule was obtained with the ‘Black’ cultivar in pot isolation. The Lara region is classified as a dried savanna with a xerophilic ecosystem in which soils are sandy with a low nutrient supply.

All root nodule homogenates from 1,212 nodules were streaked onto YMA. However, bacteria were isolated from 235 (19% of the total) nodule suspensions. Among them, 120 strains representative of each site based on their phenotypes were selected and inoculated into *P. vulgaris* ‘Tacarigua’ (Black cultivar). Only 63 isolates produced nodules (Table 1), and were subjected to a phylogenetic analysis.

### Phylogenetic analysis and distribution of Venezuelan rhizobia

**Fig. 2.**
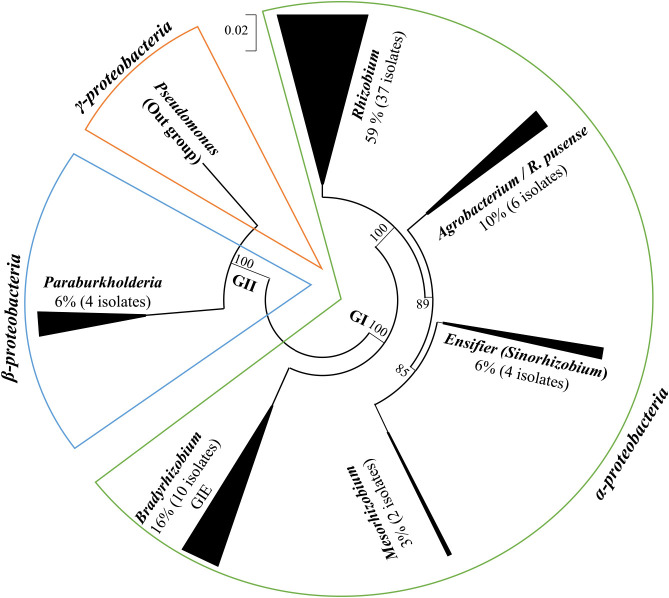
Rooted phylogenetic tree based on 16S rRNA sequences of *Phaseolus* rhizobia from different agroecosystems in Venezuela. Sixty-three isolates and 37 references strains. Numbers at the nodes indicate the level of bootstrap support (%) based on a neighbor-joining analysis of 1,000 re-sampled datasets. The scale bar was 0.02. Modeling was based on maximum likelihood and circle topology.

**Fig. 3.**
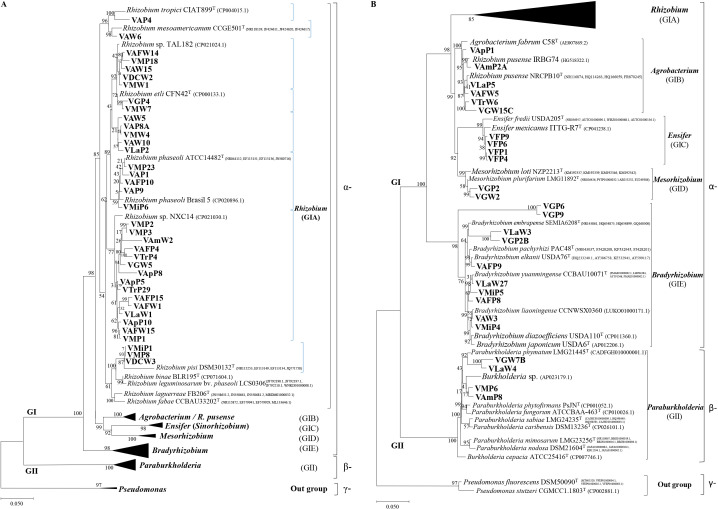
Phylogenetic analysis of Venezuelan isolates based on concatenated sequences as MLST with 16S rRNA sequences. The phylogenetic tree included *Phaseolus* rhizobia isolated in the present study (63 isolates) and references of *α-proteobacteria* and *β-proteobacteria* (37 strains). The tree is based on differences in 4,000-bp DNA fragments. The scale bar represents substitutions per nucleotide position, and each genus includes the percentage of all isolates. Numbers at the nodes indicate the bootstrap support level (%) based on a neighbor-joining analysis of 1,000 re-sampled datasets. A) Details on species in the *Rhizobium* genus with other rooted genera. B) Details on other rhizobial genera with the rooted *Rhizobium* genus. In cases in which accession numbers for genomes are not available, those for 16S rRNA, *rec*A, *atp*D, and *gln*A are shown.

MLST results obtained using the 16S rRNA gene and housekeeping genes (Fig. 3) were similar to those of the phylogenetic tree based on the 16S rRNA gene (Fig. 2). Venezuelan isolates were clustered into two bacterial groups: *α-proteobacteria* (GI) and *β-proteobacteria* (GII) with two *Pseudomonas* species in the out-group (*γ-proteobacteria*). Most of the Venezuelan isolates (94% of the total) were identified as *α-proteobacteria* (Fig. 2).

The GI cluster was divided into five sub-groups, GIA to GIE (Fig. 3A). GIA consisted of 37 Venezuelan isolates and 12 reference strains, including important Latin American strains, such as *R. mesoamericanun* CCGE501, *R. phaseoli* ATCC14482, *R. etli* CFN42, *R. tropici* CIAT899, and *R. laguerreae* FB206, and were further divided into eight sub-groups. The *Rhizobium* isolates in GIA were widely distributed in Venezuelan regions, except in Falcón (Table 2). *Rhizobium* was predominant in Mérida, DC, and Aragua without fertilizer (Table 1 and 2). GIB consisted of the reference strains *Agrobacterium fabrum* and *R. pusense* and six Venezuelan isolates from Trujillo, Amazonas, Guárico, and Lara (Fig. 3B). GIC contained two references of *Ensifer* and four Venezuelan isolates from Falcon (Table 2 and Fig. 3B). GID grouped *Mesorhizobium* reference species and two Venezuelan isolates from Guárico; this is the first study to identify *Mesorhizobium* in Venezuelan cultivars (Fig. 3B). The isolates classified as *Ensifer* and *Mesorhizobium* showed more biogeographic specificity than *Rhizobium* and *Bradyrhizobium* (Table 2). GIE consisted of ten isolates and *Bradyrhizobium* reference strains (Fig. 3B), with VLaW3 and VGP2B being closely related to *B. embrapense*, VAFP9 to *B. elkanii*, VLaW27, VMiP5, and VAFP8 to *B. yuanmingense*, and VAW3 and VMiP4 to *B. liaoningense*. The sub-clusters of *B. japonicum* and *B. diazoefficiens* did not include Venezuelan isolates (Fig. 3B). VGP6 and VGP9 showed identity with *Bradyrhizobium* sp. (91.4%) and uncultured *Bradyrhizobium* (91.3%) in the Blast search.

Group GII contained four Venezuelan isolates and reference strains of *β-proteobacteria*, such as *Burkholderia* and *Paraburkholderia* (Fig. 3B), with VGW7B and VLaW4 being related to *Paraburkholderia phymatum*. The other isolates, VMP6 and VAmP8, were classified as *Burkholderia* sp. (Table 2).

### Phylogenetic analysis based on *nod*D gene sequencing

**Fig. 4.**
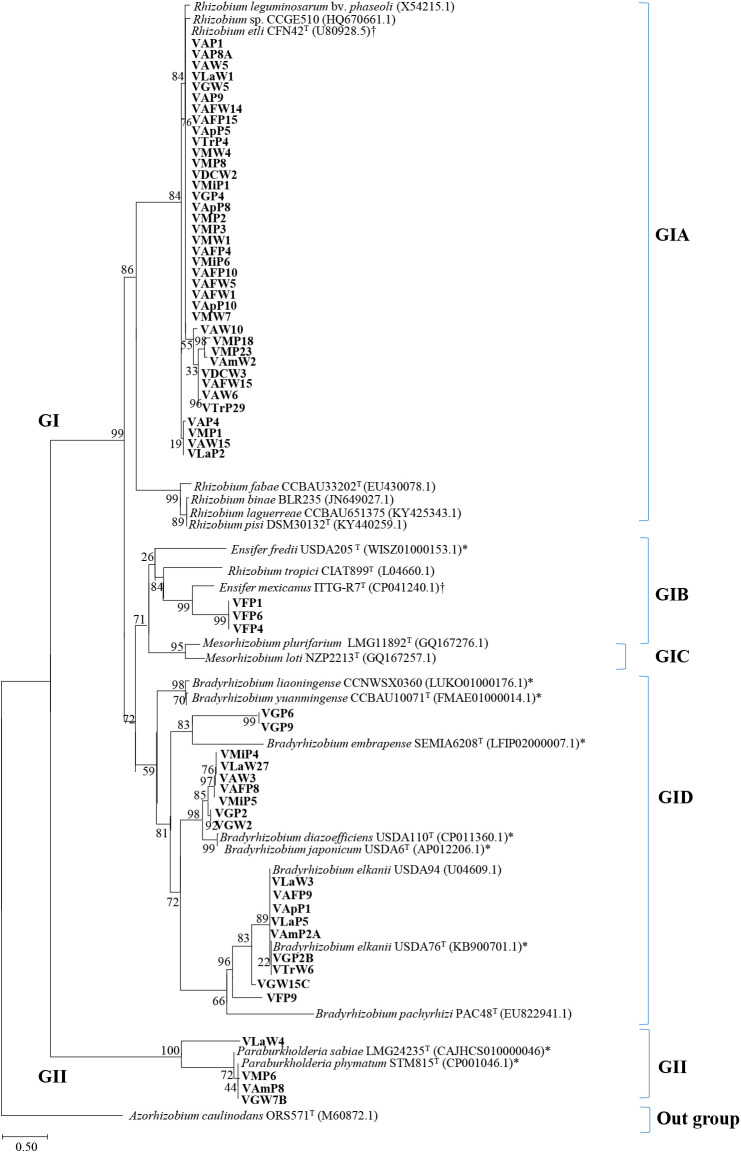
Phylogenetic tree constructed with a 690-bp DNA fragment from the *nodD* gene. The numbers at the branch nodes indicate bootstrap values (%), based on a neighbor-joining analysis of 1,000 re-sampled datasets. The scale bar indicates substitutions per site. † indicates the accession numbers of the plasmid sequences, including the *nod*D gene; * indicates the genome accession numbers, including the *nod*D genes.

To construct a phylogenetic tree based on *nod*D gene sequences, *Azorhizobium caulinodans* ORS571 was selected as the symbiotic out-group (Fig. 4). Fifty-nine isolates were grouped as GI with different genera and further sub-grouped as GIA to GID (Fig. 4). GIA consisted of 38 isolates divided as follows: 26 isolates closely related and eight isolates slightly related to *Rhizobium* sp. (HQ670661.1)/*R. etli* CFN42 (U80928.5), and four isolates not related to any reference strain. GIA isolates included VAFW5, which was in the *Agrobacterium* group based on MLST (Fig. 3B). In contrast to MLST, no isolate was classified as *R. pusense* based on *nod*D. GIB contained VFP1, VFP6, and VFP4, classified as *Ensifer* sp. in MLST, which were closely related to the *Ensifer mexicanus* reference strain based on the *nod*D gene. GIC grouped *Mesorhizobium* reference strains, and no Venezuelan isolate was included in this group.

The *Bradyrhizobium* group (GID) was divided into two sub-groups (Fig. 4). The first group contained 9 Venezuelan isolates and reference strains of *B. embrapense*, *B. yuanmingense*, *B. liaoningense*, and *B. japonicum*. VGP2 and VGW2 from Guárico, classified as *Mesorhizobium* based on MLST, were classified into the *B. japonicum* group based on the *nod*D gene sequence. VGP6 and VGP9 were also in this sub-group with the nearest relationship with *B. embrapense* SEMIA 6208. The second sub-group contained 9 Venezuelan isolates and the reference strains of *B. elkanii* and *B. pachyrhizi* (Fig. 4). Five of these isolates were related to *R. pusense* in MLST (Fig. 3B). VFP9 was close to *Ensifer* references in MLST; however, this isolate was related to *B. elkanii* based on the *nod*D gene. GII consisted of four isolates and *Paraburkholderia* reference strains (Fig. 4). These four isolates were also classified with *β-proteobacteria* in MLST (Fig. 3B). In the phylogenetic tree based on *nod*D sequences, three isolates in GII were clustered with *P. phymatum*.

### Phylogenetic analysis based on *nif*H gene sequences

**Fig. 5.**
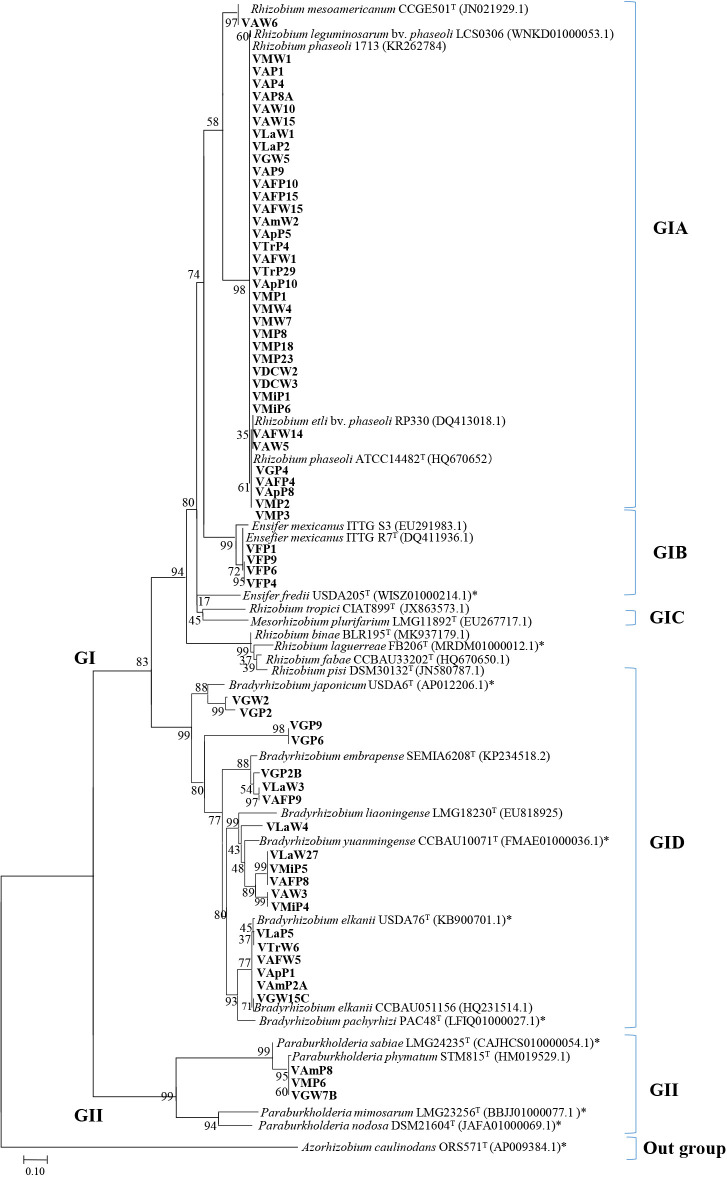
Phylogenetic tree constructed with a 750-bp DNA fragment from *nif*H gene sequences. The numbers at the branch nodes indicate bootstrap values (%) based on a neighbor-joining analysis of 1,000 re-sampled datasets. The scale bar indicates changes per site. * indicates genome accession numbers, including the *nifH* genes.

The phylogenetic tree based on *nif*H gene sequences included *A. caulinodans* ORS571 as a symbiotic out-group (Fig. 5). The classification of isolates was similar to the *nod*D gene analysis, with two groups GI and GII. GI was further divided into four sub-groups: GIA to GID. GIA contained 60% of the Venezuelan isolates and *Rhizobium* reference strains. VAW6 was classified as *R. mesoamericanum* based on the *nif*H gene (Fig. 5), similar to MLST (Fig. 3A). Thirty-six isolates were closely related to *phaseoli* reference strains based on the *nif*H gene (Fig. 5), and were further divided into three sub-clusters with four references of *phaseoli* strains. The first sub-cluster included the type strain *R. phaseoli* ATCC14482; this type strain isolated from Mexico with *Phaseolus* species was found in different American continent regions and was reported to be closely related to *R. etli* bv. *phaseoli* (Ribeiro *et al.*, 2013), which is consistent with the present results. The second sub-group included *R. etli* bv. *phaseoli* RP330 and two isolates. The last sub-group included *Rhizobium phaseoli* 1713, isolated from a non-tropical province in China with *Phaseolus* species (Wang *et al.*, 2016), and was closely related to *Rhizobium leguminosarum* bv. *phaseoli* LCS0306, a highly effective inoculant from Spain (Pastor-Bueis *et al.*, 2019).

GIB based on *nif*H consisted of *E. mexicanus* reference strains and four isolates VFP1, VFP9, VFP6, and VFP4, which were also classified as *E. mexicanus* in MLST (Fig. 3B and 5). *Mesorhizobium* references were classified as GIC without any Venezuelan isolate (Fig. 5).

GID consisted of 19 isolates and reference strains of *Bradyrhizobium* (Fig. 5) and were further sub-divided as follows. VGP2 and VGW2 were sub-grouped with the reference of *B. japonicum* based on the *nif*H gene, while these isolates were classified as *Mesorhizobium* sp. in MLST. VLaW3, VGP2B, and VAFP9 were sub-grouped with *B. embrapense* SEMIA 6208 based on the *nif*H gene. VTrW6, VApP1, VLaP5, VAmP2A, VGW15C, and VAFW5 were closely related to *B. elkanii* based on the *nif*H gene (Fig. 5), while these isolates were classified as *R. pusense* in MLST (Fig. 3B). VAW3, VMiP4, VLaW27, VAFP8, and VMiP5 were closely related to *B. yuanmingense* based on the *nif*H gene (Fig. 5), showing similar classifications to those in MLST (Fig. 3B). VGP9 and VGP6 were classified into the *Bradyrhizobium* group; however, since there was no reference strain closely related to them, they were classified as *Bradyrhizobium* sp.

The remaining isolates were in GII with strains of *β-proteobacteria* (*Paraburkholderia*) based on the *nif*H gene (Fig. 5); this is congruent with the phylogenetic analysis based on MLST and the *nod* gene. Three isolates, VAmP8, VMP6, and VGW7B, were closely related to *P. phymatum*. VLaW4, classified as *Paraburkholderia* in MLST, was grouped with *Bradyrhizobium* in GID for the *nif*H gene.

Consequently, the *Nif* assessment was based on the detection of N_2_ fixation. Several isolates did not exhibit the ability to fix N_2_ because ARA was not detected (Table 2). Ineffective isolates were mainly related to *Bradyrhizobium*, and two isolates belonged to *R. pusense* and *Ensifer*. VMP2 was classified as *Rhizobium* sp. based on MLST, and the *nod*D and *nif*H genes showed the highest ARA (89.2±3.2‍ ‍μM C_2_H_4_ h^–1^ g^–1^ dry weight of nodules).

### Physiological characterization of *Phaseolus* rhizobia under abiotic stress conditions

All 63 genetically categorized isolates were phenotypically characterized and assessed under different abiotic stress conditions (Table S1). Growth rates were classified into three groups: fast growers (24–72 h, 74% of isolates), intermediate growers (96–120 h, 24% of isolates), and slow growers (≥144 h, 2% of isolates). Isolates were classified into three types according to color: 14 were white (W), four were transparent (T), and the remaining were white-transparent (WT). According to texture, four isolates were classified as sticky (SS) and 59 as creamy (C).

Strains showed different growth abilities under high temperature conditions. A total of 99% of isolates grew at 45°C, with weaker, better, or the same growth as the control. All isolates grew at 20°C, whereas those from hot regions (Lara and Falcon) showed weak growth. VAFW14, VGP9, and VLaW3, classified as *Bradyrhizobium* in MLST, were fast growers (48 h). The majority of isolates utilized sucrose as a carbon source and exhibited the ability to live as free-living bacteria using Ashby medium, which is generally used for free-living diazotrophic bacteria (unpublished data).

Salinity tolerance was assessed by recording growth with NaCl. A total of 98% of isolates grew at low NaCl concentrations (1 and 2%) with better or the same growth as the control. Two isolates, VMiP5 from Alfisol (*B. yuanmingense*) and VTrP4 (*Rhizobium* sp.) from acidic soil (Ultisol), were not tolerant of NaCl at 3 or 4%. The growth of isolates from Apure, Guárico, and Falcon was not affected by NaCl, even at 4%. Four isolates obtained from Falcon (Aridisol), 14 out of 15 isolates from Guárico or Lara (Vertisol), and two from Andes (Ultisol) showed high salinity tolerance growing at 4% of NaCl (Table S1).

Venezuelan isolates showed tolerance to different pH conditions from acidic to alkaline; however, their growth was inhibited more by acidic than alkaline conditions. Among the isolates that mainly originated from alkaline soils, such as Falcon, Guárico, and Aragua (without fertilizer), 18% did not grow at pH 4.5, and VTrP4 (*Rhizobium* sp.) did not grow even at pH 5.0. In contrast, all isolates survived under alkaline conditions at pH 10, with only 2% showing weak growth (Table S1).

The effects of Al on bacterial growth were recorded under two pH conditions. All isolates, except for VTrP4, survived with 2‍ ‍mM Al at neutral pH. In contrast, only 8% of isolates grew under 2‍ ‍mM at pH 4.5 (Table S1). Among isolates from soils with neutral or alkaline pH, such as Aragua (without fertilizer), Guárico, and Falcon, growth was severely inhibited by 2‍ ‍mM of Al at pH 4.5.

The antibiotic resistance profiles of isolates were examined against the following antibiotics, spectinomycin (Spe), streptomycin (Str), kanamycin (Kan), nalidixic acid (Nal), and chloramphenicol (Cp). Some isolates exhibited multi-tolerance, and VAFP4, VAFW1, VAW10, VAW6, VMP3, and VLaW3 exhibited resistance to all antibiotics (Table S1). A total of 90% of isolates were resistant to Nal. In contrast, the growth of 86% of isolates was strongly inhibited by Str. Most isolates obtained from Lara, Amazonas, Trujillo, Miranda, and Aragua (without fertilization) were sensitive to Str.

No relationship was observed between antibiotic resistance and pH or Al tolerance (*P*≥0.05). VTrP4 (*Rhizobium* sp.) was susceptible to all antibiotics. Furthermore, it did not survive under low pH and high Al. In contrast, VGP6 and VGP9, classified as *Bradyrhizobium* sp., showed high tolerance to almost all abiotic stress conditions. Other isolates with high performance under abiotic stress were VAP4, VApP10, VApP8, VMP23 (classified as *Rhizobium*), and VGW2 (*Mesorhizobium* sp.) (Table S1).

### Plant growth-promoting profiles of Venezuelan isolates

A summary of symbiotic performance, physiological profiles as plant partners, antifungal profiles, and phylogenetic groups based on MLST are shown in Table 2. Physiological profiles as plant partners included activities directly or indirectly related to plant growth, such as IAA, P, or K solubilization. Six isolates significantly increased plant biomass with ≥2,000‍ ‍mg plant^–1^, and were classified as *Rhizobium* (VMiP1, VApP5, VAmW2, VTrW6, and VMP2), except for VAmP8, which was classified as *Burkholderia* in MLST. Auxin hormone production was dominated by *Rhizobium* (VAFP10, VAP4, VMP1, VMP8, and VLaP2) and *Mesorhizobium* (VGP2) isolates with more than 90‍ ‍μg mL^–1^. Fourteen isolates did not produce detectable levels of IAA, but exhibited strong antifungal activity (Table 2). Isolates with the best performance were as follows: VMP1 (*Rhizobium* sp.) for IAA (198.99‍ ‍μg mL^–1^), VFP6 (*Ensifer* sp.) for P solubilization (1.67 index), and VMP23 (*R. phaseoli*) for K solubilization (1.22 index). Among all isolates, only VGP4 (*Rhizobium* sp.) and VFP6 (*Ensifer* sp.) showed Fe chelation (by CAS media, unpublished data).

Furthermore, 46% of isolates exhibited antifungal activity against at least one pathogen at different levels (Table 2). Twenty-five isolates exhibited activity against *P. aphanidermatum*, whereas only ten isolates exhibited activity against *C. gloeosporioides*. VTrP29 (*Rhizobium* sp.) showed good responses against all fungal pathogens, while VGP9 (*Bradyrhizobium* sp.) exhibited very high potential against all tested pathogens, mainly *P. aphanidermatum*, *R. necatrix*, and *H. mompa*. VTrP4 (*Rhizobium* sp.) exhibited activity against four pathogens (*P. aphanidermatum*, *R. solani*, *P. oryzae*, and *R. necatrix*). These isolates with high antifungal performance exhibited low physiological activities for plant growth promotion and symbiotic performance.

## Discussion

### Genetic distribution and relationship of symbiotic genes

The diversity of rhizobia isolated from *P. vulgaris* has been examined worldwide with various techniques and criteria. Collectively, the present results indicate that many species nodulate Venezuelan common beans; these cultivars were promiscuous hosts. Bacterial cells were only obtained from 19% of the 1,212 nodules collected in the present study (Table 1), indicating that many were unculturable. Furthermore, only 63 out of 120 strains produced nodules when inoculated on the Black cultivar, which may have been due to the specificity of the isolates to *Phaseolus* species because we only used one type of cultivar to corroborate nodulation activity. *Phaseolus* rhizobia were widely distributed in Venezuela, including areas without its cultivation history. The highest number of isolates was found in an area with the soil type Inceptisol, which may be because beans have been continuously cultivated in areas with Inceptisol, such as in Aragua (Fig. 1, Table 1).

The geographical distribution and diversity of rhizobia are mainly influenced by the climate and distribution of host legumes; however, other factors in soil parameters, such as pH, salinity, and nutrient content, are important because they affect the distribution of host legumes (Córdova-Sánchez *et al.*, 2011; Chidebe *et al.*, 2018). Graham (1992) reported that crop production and rotation, soil pH, and other factors may affect rhizobial diversity and occupancy. The present results demonstrated that legumes belonging to the *Phaseolus* genus were nodulated by rhizobia across *α-* and *β-proteobacteria*, which is consistent with the findings of Andrews and Andrews (2017). However, in most studies, *Phaseolus* symbionts were reported separately with a maximum of one or two genera. The present study is the first wide exploration of rhizobia associated with two different *P. vulgaris* cultivars that are endemic in Venezuela. The results obtained showed that rhizobial diversity associated with *P. vulgaris* distributed in one country, Venezuela, was greater than in other countries, such as Spain, Brazil, Ecuador, and Mexico (Velázquez *et al.*, 2005; Zurdo-Piñeiro *et al.*, 2009; Ribeiro *et al.*, 2013; Verástegui-Valdés *et al.*, 2014).

In the present study, a larger number of Venezuelan isolates associated with *Phaseolus* were *Rhizobium* species with predominance related to *R. phaseoli* (Fig. 3A) than in other countries in which *R. tropici* or *R. etli* bv. *phaseoli* were predominantly associated with *Phaseolus* species (Martínez-Romero *et al.*, 1991; Eardly *et al.*, 1995; De Oliveira-L *et al.*, 2015). Isolates classified as *R. phaseoli* in the present study were heterogeneous because they showed different stress tolerance (Crow *et al.*, 1981; Wang *et al.*, 2016; Mwenda, 2017). Previous studies reported that when *R. etli* bv. *phaseoli* populations were low, there were high numbers of rhizobia other than *R. etli* in bean nodules (Martinez-Romero, 2003; Zurdo-Piñeiro *et al.*, 2009; Andrews and Andrews, 2017), which is consistent with the present results.

We identified *Agrobacterium/R. pusense* as nodulating bacteria in *Phaseolus* (Fig. 3B), which is consistent with previous findings (Velázquez *et al.*, 2005; Ribeiro *et al.*, 2013; Verástegui-Valdés *et al.*, 2014).

*Ensifer* is rarely reported to nodulate *Phaseolus* species. In contrast, in the present study, strains classified as *Ensifer* were isolated from Falcon with the arid ecosystem and alkaline pH, nearest to the coast (Table 2), which is in accordance with findings from Tunisia, Mexico, and China (Mnasri *et al.*, 2007; Zurdo-Piñeiro *et al.*, 2009; Mnasri *et al.*, 2012; Verástegui-Valdés *et al.*, 2014; Mhamdi *et al.*, 2015; Wang *et al.*, 2016).

We detected two *Mesorhizobium* isolates from Guárico (Vertisol) with low nutrients, such as N and P, which was similar to the distribution of *Mesorhizobium* in soils with low nutrients in Brazil (Grange and Hungria, 2004). However, *Mesorhizobium* has been more commonly associated with *Astragalus* and *Glycyrrhiza* than *P. vulgaris* (Andrews and Andrews, 2017).

In the present study, the isolates classified as *Bradyrhizobia* in MLST were also capable of nodulating *Phaseolus*, which is supported by other findings on isolates from Mexican, Peruvian, and Brazilian soils (Barcellos *et al.*, 2007; López-López *et al.*, 2013; Matsubara and Zúñiga-Dávila, 2015). Since *P. vulgaris* from the tribe *Phaseoleae* is indigenous to the American continent (principally South and Central America), the nodulation of *Bradyrhizobium* species in *P. vulgaris* may be ascribed to the adaptation of legumes in Venezuela (Wang *et al.*, 2016). It may also be due to adaptations from inoculants applied through the seeds in other legumes, such as soybean (Barcellos *et al.*, 2007; Nandasena *et al.*, 2007; López-López *et al.*, 2013; Matsubara and Zúñiga-Dávila, 2015).

We also identified isolates that are closely related to *Burkholderia* with nodulating ability on *P. vulgaris*, which is consistent with previous findings on isolates from Moroccan and Brazilian soils (Talbi *et al.*, 2010; Ferreira *et al.*, 2012; Dall’Agnol *et al.*, 2016); however, the predominant species of *Burkholderia* differed in the present study. Our results are consistent with previous findings on isolates from Latin American countries, such as Mexico, Venezuela, and Uruguay, in which *Burkholderia* was mainly associated with *Mimosa* species (Chen *et al.*, 2005; Lammel *et al.*, 2013; Bontemps *et al.*, 2016; Platero *et al.*, 2016). In the present study, species of *β-proteobacteria* were observed in nutrient-deficient soils, such as Guárico, Lara, and Amazonas. *Burkholderia* is reportedly distributed to infertile acidic and low nutrients soils (Graham, 1992; De Oliveira-L *et al.*, 2015; Bontemps *et al.*, 2016; Platero *et al.*, 2016), and this also appears to be the case in Venezuela.

Strong correlations were observed between soil types and rhizobia types; *Rhizobium* predominantly correlated with soil types such as Ultisol (Trujillo and Mérida), Alfisol (Miranda and D.C), Inceptisol (Aragua), and Oxisol (Amazonas). However, several isolates belonging to *Burkholderia* were also found in Mérida and Amazonas (Table 2 and S1). *Ensifer* strains were only found in Falcón with Aridisol. In contrast, highly diverse groups were detected in Guárico and Lara with Vertisol, such as *Bradyrhizobium*, *Rhizobium*, *Mesorhizobium*, and *Burkholderia*.

In the present study, all four strains classified as *β-proteobacteria* in MLST had *nod*D genes closely related to *Paraburkholderia* (Fig. 4), suggesting that these genes in the four strains originated from *P. phymatum*. Our results on the *nod*D sequences and nodulation activity for *β-proteobacteria* are similar to the findings of other studies on isolates from Brazilian forests and Moroccan soils, describing the symbiotic effectiveness of *β-proteobacteria* with common beans (Talbi *et al.*, 2010; Lammel *et al.*, 2013).

VGP2 and VGW2 from Guárico classified as *Mesorhizobium* based on MLST were closely related to *B. japonicum* based on the *nod*D gene sequence (Fig. 4), suggesting the horizontal gene transfer of symbiotic islands in these strains. The horizontal gene transfer of the *nod*D gene between *Mesorhizobium* and *Bradyrhizobium* has also been reported (Sullivan and Ronson, 1998; Grange and Hungria, 2004; Barcellos *et al.*, 2007; Nandasena *et al.*, 2007). Furthermore, the present results suggest the transfer of the *nod*D gene from *Bradyrhizobium* into *Ensifer*, *Mesorhizobium*, and *R. pusense* isolates, which may be from *Bradyrhizobium* inoculants applied through seeds in other legumes, such as soybean.

The present results also indicate the transfer of *nif*H genes from *Bradyrhizobium* into *Rhizobium*. *nif*H genes are regarded as markers of the efficiency of N_2_ fixation. In the present study, strains with the *nif*H gene classified as *Bradyrhizobium* showed slightly low nodulation, and ARA resulted in a low biomass in plants, such as VAFP9 and VLaW3 with no detectable ARA (Table 2). VLaW4 classified as *Burkholderia* in MLST was grouped with *Bradyrhizobium* for the *nif*H gene, suggesting horizontal gene transfer between different *proteobacteria* groups. The effectiveness of nodulation may correlate with their origin, cultivation conditions, rhizobia type, and gene transfer, as previously reported (Vargas and Graham, 1988; Martínez-Romero *et al.*, 1991; Hungria *et al.*, 1993; Graham *et al.*, 1994; Eardly *et al.*, 1995; Chidebe *et al.*, 2018).

### Physiological characteristics and stress tolerance of *Phaseolus* rhizobia

The abilities of tropical rhizobia other than N_2_ fixation currently remain unclear; therefore, the present results add new and essential information on the characteristics of rhizobia distributed in tropical areas. Most of the strains isolated in the present study not only survived, they also adapted well to abiotic stress conditions, such as acidic soils, high temperatures, and high salinity. Previous studies support this result, showing that the most tolerant isolates were *α-proteobacteria* and their tolerance depended on their soil of origin (Graham *et al.*, 1994; Martinez-Romero, 2003; Marquina *et al.*, 2011; Mhamdi *et al.*, 2015). The present results indicate that Venezuela possesses a great diversity of rhizobia that may be beneficial for agriculture under stress conditions.

In the present study, the strains isolated from soils with Al or acidic pH showed higher tolerance to Al-acidic conditions (Table S1), suggesting that the tolerance of isolates is associated with soil origin and cultivation history, as reported by Piña and Cervantes (1996). Low soil pH is often attributed to Al and Mn toxicity and Ca deficiency (Graham *et al.*, 1981; Graham *et al.*, 1994), and to improve soil conditions, calcium carbonate was added before and during *Phaseolus* cultivation for the traditional cropping system (Casanova, 2005) in some sampling sites in Venezuela, such as Trujillo. Many studies describe *Rhizobium* species showing resistance to acidic pH (*e.g.*, *R. tropici*) (Muglia *et al.*, 2007; Marquina *et al.*, 2011), while *Rhizobium* species sensitive to low pH have also been reported, including *R. meliloti* (Tiwari *et al.*, 1992). Acidic pH tolerance in rhizobia depends on the maintenance of intracellular pH (Graham *et al.*, 1994).

Furthermore, most of our isolates were tolerant to alkaline pH, which is supported by previous findings showing that *R. phaseoli* strains were more competitive than other* Rhizobium* species under alkaline conditions (Shamseldin and Werner, 2004; Verástegui-Valdés *et al.*, 2014).

Additionally, antifungal activity results (Table 2) suggest that strains have the ability to promote plant health based on their resistance to pathogens. Several isolated strains did not show good potential as inoculants due to low IAA, P, and K; however, these rhizobia exhibited strong antifungal activity against different fungi, which may suppress the disease activity of soil-borne pathogens and reduce disease severity.

VGP6 and VGP9 were genetically unrelated to any reference strains, suggesting that these isolates belong to the *Bradyrhizobium* group. According to the MLST, *nodD*, and *nifH* sequence analyses, these may be novel species or lineages that are fast or intermediate growers. VGP6 and VGP9 may improve biomass, show tolerance to different abiotic stress conditions, and exhibit antifungal activity.

Stress tolerance and physiological abilities in rhizobia isolated from specific areas may be attributed to wild *Phaseolus* rhizobia. Wild common beans have been reported from Mérida, Portuguesa, Táchira, and Trujillo in the western Andes of Venezuela and some Lara areas (Debouck *et al.*, 1993). Diverse rhizobia with tolerance to stress in these areas may be associated with the native wild legumes.

More factors affected the ability of rhizobia to promote plant growth than N_2_ fixation. Our plant assay was performed at a neutral pH and under controlled temperature, light, and humidity; however, in fields with various stressors, isolated rhizobia with stress tolerance may help plants to grow better. Further studies are needed to confirm the effectiveness of strains as inoculants for *Phaseolus* in field conditions with acidic soils, high Al concentrations, low nutrient supply, or the presence of pathogens.

## Citation

Ramírez, M. D. A., España, M., Sekimoto, H., Okazaki, S., Yokoyama, T., and Ohkama-Ohtsu, N. (2021) Genetic Diversity and Characterization of Symbiotic Bacteria Isolated from Endemic *Phaseolus* Cultivars Located in Contrasting Agroecosystems in Venezuela. *Microbes Environ ***36**: ME20157.

https://doi.org/10.1264/jsme2.ME20157

## Supplementary Material

Supplementary Material

## Figures and Tables

**Table 1.** General characteristics of soils and sites of plant samples

**Isolation**	**Origin state** (Sites)	**Cardinal location**	**Ecosystem**	**Soil type**	**Vegetation**	**Legume cultivation history**	**Number of nodules detached from** ***P. vulgaris***				**Number of strains**		
							by cultivar		Total		Obtained	Selected	**Clarified**
							Black	White					
**Nodules from soils inoculated in pot cultivation**	**Amazonas**	South	Jungle/rain forest	Oxisol^††^	Cucumber, tomato, coriander, *Capsicum* sp.	—	30	26	56		12	6	**3**
	**Apure**	Southwest	Floodplain	Inceptisol	*Acacia* sp., *Caraipa* sp., *Mauritia* sp. several trees	*Phaseolus vulgaris*^†^	32	6	38		10	5	**4**
	**Aragua**	North-central	Valley, without fertilizer	Inceptisol	*Zea maize, grasses, Fabaceae*	*Arachis* sp., *Phaseolus* species	42	50	92		48	24	**6**
			Valley with Fertilizer		*Asteracea* sp., grasses	*Phaseolus* species., *G. max*, *Vigna* spp.	130	90	220		27	13	**5**
	**DC (Caracas)**	North-central	N. Park on City	Alfisol	*Coffea* sp., sugarcane, forestal species, *Bryophytes*	—	0	23	23		5	3	**2**
	**Falcón**	Northwest	Aridic, xerophilic ecosystem	Aridisol	Mining and *Prosopis* sp., *Opuntia* sp.	—	60	30	90		13	7	**4**
	**Guárico**	Central	Savanna	Vertisol	Species of grasses	*Phaseolus vulgaris*^†^	93	61	154		14	7	**5**
	**Lara**	West-central	Dried savanna	Vertisol	*Coffea* sp., *Inga* sp., grasses.	—	0	77	77		10	5	**4**
	**Mérida**	Southwest	Andes-Temperate	Ultisol	*Theobroma* sp., *Musa* sp., *Lactuca* sp., *Mimosa*	—	130	80	210		31	16	**10**
	**Miranda**	Central coast	Mountain	Alfisol	Forestal species (*Mimosa* sp.), *Pseudobombax* sp.	*Phaseolus* species ^†^	18	9	27		17	9	**4**
	**Trujillo**	Northwest	Andes	Ultisol	*Coffea* sp., *Mimosa* sp., *Caesalpiniaceae*	*Phaseolus* species ^†^	47	5	52		5	3	**3**
**Nodules from fields in Venezuela**	**Aragua**	North-central	Valley, without fertilizer	Inceptisol	*Corn*, grasses, *Sorghum* sp.	*Phaseolus* species	20	3	23		22	11	**3**
			Valley, with fertilizer	Mollisol	Cereals, grasses, and forestal trees	*Phaseolus vulgaris*	63	10	73		12	6	**4**
	**Lara**	West-central	Dried savanna	Vertisol	*Coffea* sp., *Inga* sp., grasses.	*Phaseolus vulgaris*	35	35	70		4	2	**2**
	**Guárico**	Central	Savanna	Vertisol	Species of grasses	*Phaseolus vulgaris*	0	7	7		5	3	**4**
Total							700	512	1,212		235	120	**63**

^††^ Amazonas soil is classified into Entisol and Oxisol; however, the sampling site was Oxisol. ^†^ *P. vulgaris* cultivars differ from those used in the present study.

**Table 2.** Summary of physiological activities as plant partners and antifungal activities of Venezuelan *Phaseoli* rhizobia

**Isolate name^†^**	**Origin** (Sites)	**MLST**	**Biomass^a^** (DW mg plant^–1^)	**Physiological activities**					**Antifungal activities^c^**						
				**ARA^b^** **(μmol C_2_H_4_ h^–1^ g^–1^ nodule DW)**	**IAA** (μg mL^–1^)	**Pi solubilizing index**	**K solubilizing index**		***Pythium aphanidermatum*** (%)	***Rhizoctonia solani*** (%)	***Fusarium graminearum*** (%)	***Pyricularia oryzae*** (%)	***Colletotrichum gloesporioides*** (%)	***Rosellinia necatrix*** (%)	***Helicobasidium mompa*** (%)
**VDCW2**	DC	*Rhizobium* sp.	809.0±30.0*	37.9±7.9	0.43	—	—		55.0	50.0	—	3.0	—	25.0	6.0
**VDCW3**		*R. pisi*	1,519.0±168.0*	86.1±4.6	45.80	0.44	0.25		53.0	25.0	2.0	—	—	80.0	100.0
**VMiP1**	Miranda	*R. pisi*	2,318.3±200.0****	1.8±0.4	36.59	0.02	—		100.0	7.0	10.0	—	—	1.0	5.0
**VMiP4**^†^		*B. liaoningense*	773.7±93.5*	0.6±0.1	10.63	0.03	—		—	—	—	—	—	—	—
**VMiP5**		*B. yuanmingense*	749.0±100.0	8.4±2.9	3.20	—	—		—	—	—	—	—	—	—
**VMiP6**		*R. phaseoli*	628.7±26.6	55.6±5.5	—	0.50	—		—	—	—	—	—	—	—
**VFP1**	Falcón	*Ensifer* sp.	1,957.0±248.0*	10.9±1.4	46.44	0.60	—		100.0	3.0	5.0	—	—	51.0	20.0
**VFP4**		*Ensifer* sp.	1,559.3±100.6*	26.1±4.2	20.48	0.20	—		—	—	—	—	—	—	—
**VFP6**		*Ensifer* sp.	1,186.5±171.2*	1.9±0.4	18.46	1.67	0.25		—	—	—	—	—	—	—
**VFP9**		*Ensifer* sp.	410.0±25.0	not detected	27.64	0.02	0.22		—	—	—	—	—	—	—
**VAFP10**	Aragua with Fertilizer	*R. phaseoli*	1,231.3±108*	3.24±1.0	90.36	—	0.75		1.0	1.0	11.0	—	—	55.0	4.0
**VAFP15**		*Rhizobium* sp.	1,776.4±80.2*	1.96±0.9	75.52	0.63	0.20		1.0	1.0	11.0	—	6.0	11.0	—
**VAFP4**^†^		*Rhizobium* sp.	845.7±71.2*	1.28±2.0	—	0.56	—		50.0	1.0	2.0	—	—	60.0	55.0
**VAFP8**^†^		*B. yuanmingense*	1,133.3±114.0*	2.4±0.4	0.57	—	0.20		50.0	10.0	1.0	50.0	—	55.0	100.0
**VAFP9**		*B. elkanii*	337.0±60.0	not detected	54.48	0.03	—		—	—	—	—	—	—	—
**VAFW1**^†^		*Rhizobium* sp.	1,636.7±118.3*	19.1±5.2	17.12	0.03	—		50.0	—	—	6.0	3.0	55.0	55.0
**VAFW14**^†^		*Rhizobium* sp.	1,587.0±5.0***	4.4±0.1	—	—	—		50.0	—	—	3.0	100.0	51.0	100.0
**VAFW15**		*Rhizobium* sp.	1,950.7±61.3*	25.7±0.3	34.55	1.50	—		—	—	—	—	—	—	—
**VAFW5**		*R. pusense*	529.1±55.1	not detected	4.11	—	—		50.0	10.0	6.0	50.0	—	55.0	100.0
**VAP1**	Aragua without Fertilizer	*R. phaseoli*	570.7±28.9	1.3±0.2	50.77	0.40	0.14		100.0	1.0	4.0	—	—	50.0	3.0
**VAP4**		*R. tropici*	1,314.7±66.0*	7.0±3.0	115.55	0.43	0.17		50.0	1.0	1.0	11.0	—	—	100.0
**VAP8A**		*Rhizobium* sp.	614.6±20.0	84.4±39.9	16.73	0.30	—		52.0	7.0	2.0	55.0	—	6.0	100.0
**VAP9**^†^		*R. phaseoli*	929.0±112.7*	60.4±4.8	22.60	0.20	—		55.0	4.0	—	—	—	4.0	100.0
**VAW10**		*Rhizobium* sp.	899.3±86.4*	32.8±16.4	4.23	—	—		2.0	6.0	21.0	51.0	—	70.0	6.0
**VAW15**		*Rhizobium* sp.	1,331.9±99.8*	18.8±1.2	—	—	—		6.0	6.0	6.0	2.0	—	11.0	—
**VAW3**^†^		*B. liaoningense*	962.5±22.5**	38.6±1.0	—	—	—		51.0	2.0	6.0	—	—	60.0	100.0
**VAW5**		*Rhizobium* sp.	1,301.0±138.0*	14.5±4.4	34.68	0.44	0.20		100.0	—	—	—	—	50.0	—
**VAW6**^†^		*R. mesoamericanum*	575.5±88.0	1.9±0.5	—	—	0.20		50.0	—	—	2.0	6.0	51.0	51.0
**VApP1**	Apure	*Rhizobium* sp.	769.3±152.7*	3.1±1.0	10.70	0.71	—		—	—	—	—	—	—	—
**VApP10**		*Rhizobium* sp.	1,620.4±290*	7.2±5.7	29.23	0.58	—		—	—	—	—	—	—	—
**VApP5**		*Rhizobium* sp.	2,050.7±183.0*	29.4±2.8	19.01	0.60	0.60		—	—	—	—	—	—	—
**VApP8**		*Rhizobium* sp.	1,642.3±76.3*	2.8±1.4	9.65	0.67	0.40		—	—	—	—	—	—	—
**VAmP2A**	Amazonas	*Rhizobium* sp.	1,860.6±108.0*	25.7±1.1	—	0.40	0.75		—	—	—	—	—	—	—
**VAmP8**		*Burkholderia* sp.	2,018.9±83.0*	77.8±4.1	40.63	0.75	0.67		—	—	—	—	—	—	—
**VAmW2**		*Rhizobium* sp.	2,012.4±147.0*	4.3±3.2	5.87	0.55	0.20		—	—	—	—	—	—	—
**VMP1**	Mérida	*Rhizobium* sp.	1,698.6±200.0*	6.9±0.8	198.99	0.33	0.14		—	—	—	—	—	—	—
**VMP18**		*Rhizobium* sp.	1,709.5±177.8***	38.3±6.1	0.39	0.75	—		—	—	—	—	—	—	—
**VMP2**		*Rhizobium* sp.	2,326.7±200.0*	89.2±3.2	30.46	0.33	0.33		100.0	—	—	—	—	—	—
**VMP23**		*R. phaseoli*	1,609.3±57.4***	1.6±0.7	7.69	—	1.22		60.0	6.0	—	—	—	60.0	—
**VMP3**		*Rhizobium* sp.	778.0±20.0*	0.6±0.2	25.85	0.03	0.20		—	—	—	—	—	—	—
**VMP6**		*Burkholderia* sp.	601.0±1.0	0.6±0.2	40.54	0.40	—		—	—	—	—	—	—	—
**VMP8**		*R. pisi*	1,554.5±160.5*	9.4±0.7	100.91	0.50	0.20		—	—	—	—	—	—	—
**VMW1**		*Rhizobium* sp.	1,982.5±78.5*	23.0±5.1	74.54	—	0.22		—	—	—	—	—	—	—
**VMW4**		*Rhizobium* sp.	1,147.4±170.5*	14.4±6.9	9.06	0.17	0.17		—	—	—	—	—	—	—
**VMW7**		*Rhizobium etli*	1,210.0±52.9*	44.7±4.2	4.48	—	—		—	—	—	—	—	—	—
**VTrP29**	Trujillo	*Rhizobium* sp.	1,034.7±20.3*	0.1±0.05	—	—	—		15.0	40.0	30.0	70.0	40.0	100.0	100.0
**VTrP4**		*Rhizobium* sp.	1,696.0±180.0*	0.4±0.1	—	0.03	—		50.0	50.0	—	2.0	—	53.0	—
**VTrW6**		*R. pusense*	2,249.2±80.0*	38.5±3.8	37.59	0.38	—		—	—	—	—	—	—	—
**VGP2**	Guárico	*M. plurifarium*	1,748.5±381.5*	22.2±5.5	97.43	0.02	—		—	—	—	—	—	—	—
**VGP2B**		*B. embrapense*	1,246.7±81.9*	1.8±0.3	—	—	—		—	—	—	50.0	3.0	—	—
**VGP4**^†^		*Rhizobium etli*	1,527.7±200*	10.5±1.7	26.04	0.40	0.67		—	—	—	—	—	—	—
**VGP6**		*Bradyrhizobium* sp.	1,519.5±186*	1.2±0.6	—	—	—		50.0	—	21.0	7.0	2.0	—	—
**VGP9**		*Bradyrhizobium* sp.	874.7±70.9*	0.4±0.3	29.51	0.50	—		51.0	1.0	2.0	25.0	21.0	55.0	100.0
**VGW15C**^†^		*R. pusense*	738.5±61.5	7.3±1.5	—	—	—		—	—	—	—	—	55.0	22.0
**VGW2**^†^		*M. plurifarium*	1,454.0±37.0*	33.5±8.9	6.08	0.86	—		50.0	5.0	—	—	100.0	51.0	100.0
**VGW5**		*Rhizobium* sp.	358.0±73.5	not detected	—	—	—		50.0	—	2.0	30.0	—	55.0	100.0
**VGW7B**^†^		*P. phymatum*	593.0±1.0	0.1±0.08	5.41	0.04	—		50.0	—	—	6.0	—	—	100.0
**VLaP2**	Lara	*Rhizobium* sp.	1,321.5±86.5*	32.1±11.9	131.17	—	0.40		—	—	—	—	—	—	—
**VLaP5**^†^		*R. pusense*	1,614.3±50.6*	1.3±0.5	—	—	0.25		60.0	2.0	7.0	3.0	—	60.0	10.0
**VLaW1**		*Rhizobium* sp.	863.3±41.6*	3.0±1.2	22.41	0.50	0.75		—	—	—	—	—	—	—
**VLaW27**^†^		*B. yuanmingense*	741.6±78.6	not detected	2.92	—	—		0.1	3.0	—	4.0	10.0	100.0	100.0
**VLaW3**		*B. embrapense*	360.5±5.0	not detected	14.27	0.33	—		—	—	—	—	—	—	—
**VLaW4**		*P. phymatum*	1,652.0±100.0*	0.8±0.1	33.23	—	—		—	—	—	—	—	—	—

All names included sampling sites and hosts, *e.g.*, VTrW6, V (Venezuela)—Tr (Trujillo)—W (white cultivar); VGP2, V (Venezuela)—G (Guárico)—P (black cultivar), ^†^ These strains were isolated from the field. In MLST, B.: *Bradyrhizobium.* R.: *Rhizobium.* P.: *Paraburkholderia.* M.: *Mesorhizobium.* (—) means no growth or activity. The plant test was performed with *Phaseolus vulgaris* ‘Black’. ^a^ Means+standard deviations with 3 biological replicates are shown. Asterisks indicate significant differences from the non-inoculated control (360.0±7.5 DW mg plant^–1^) in Dunnett’s test. (*P<0.05, **P<0.01, ***P<0.001, *** *P*<0.0001). ^b^ Means+standard deviations with 3 biological replicates are shown. ^c^ The numbers in strains exhibiting strong antifungal activities are highlighted.
