# Supplementary material for: Genetic Diversity and Characterization of Symbiotic Bacteria Isolated from Endemic Phaseolus Cultivars Located in Contrasting Agroecosystems in Venezuela
Source: Microbes Environ. 2021 Jun 5;36(2):ME20157. doi: 10.1264/jsme2.ME20157 (PMC8209454; doi:10.1264/jsme2.ME20157)
Supplement: Supplementary file 1 — Supplementary Material [file 36_20157_s1.pdf]

Table S1. Phenotypical and tolerance profile of Venezuelan *Phaseoli* strains under different stress conditions.

Isolate name	Origin			Isolation	MLST	16S rRNA	Phenotype			Abiotic stress						Antibiotic Profile				
	Sites	Soil type	Host cultivar				Growth rate	Color	Texture	Temperature °C	pH		NaCl %	$AlCl_3$ (mmol L^{-1})		Spe	Str	Kan	Nal	Cp
											Acid	Alkaline		pH 6.8	pH 4.5	40 μg mL^{-1}	40 μg mL^{-1}	30 μg mL^{-1}	30 μg mL^{-1}	80 μg mL^{-1}
**VDCW2**	DC	Alfisol	White	Pot	*Rhizobium* sp.	α-proteobacteria	48	WT	C	20 - 45	4.5 - 5	8 - 10	1 - 4	0.1 - 2	0.1 - 0.5	R	S	S	R	R
**VDCW3**		Alfisol	White	Pot	*R. pisi*	α-proteobacteria	48	WT	C	20 - 45	4.5 - 5	8 - 10	1 - 4	0.1 - 2	0.1 - 0.5	R	S	S	R	R
**VMiP1**	Miranda	Alfisol	Black	Pot	*R. pisi*	α-proteobacteria	48	WT	C	20 - 45	4.5 - 5	8 - 10	1 - 4	0.1 - 2	0.1 - 0.5	S	S	S	R	R
**VMiP4**		Alfisol	Black	Pot	*B. liaoningense*	α-proteobacteria	72	WT	C	20 - 45	4.5 - 5	8 - 10	1 - 4	0.1 - 2	0.1	R	S	R	R	R
**VMiP5**		Alfisol	Black	Pot	*B. yuanmingense*	α-proteobacteria	72	WT	C	20 - 40	4.5 - 5	8 - 10	1 - 2	0.1 - 2	0.1 - 0.5	S	S	S	R	R
**VMiP6**		Alfisol	Black	Pot	*R. phaseoli*	α-proteobacteria	48	WT	C	20 - 45	4.5 - 5	8 - 10	1 - 4	0.1 - 2	0.1 - 0.5	S	S	S	R	S
**VFP1**	Falcón	Aridisol	Black	Pot	*Ensifer* sp.	α-proteobacteria	48	WT	C	20 - 45	5	8 - 10	1 - 4	0.1 - 2	-	S	S	S	R	S
**VFP4**		Aridisol	Black	Pot	*Ensifer* sp.	α-proteobacteria	48	WT	C	20 - 45	5	8 - 10	1 - 4	0.1 - 2	0.1	S	S	S	R	R
**VFP6**		Aridisol	Black	Pot	*Ensifer* sp.	α-proteobacteria	48	WT	C	20 - 45	4.5 - 5	8 - 10	1 - 4	0.1 - 2	-	S	S	S	R	S
**VFP9**		Aridisol	Black	Pot	*Ensifer* sp.	α-proteobacteria	48	WT	C	20 - 45	5	8 - 10	1 - 4	0.1 - 2	-	S	S	S	R	S
**VAFP10**	Aragua with Fertilizer	Inceptisol	Black	Pot	*R. phaseoli*	α-proteobacteria	72	WT	C	20 - 45	4.5 - 5	8 - 10	1 - 4	0.1 - 2	0.1 - 0.5	S	S	S	R	R
**VAFP15**		Inceptisol	Black	Pot	*Rhizobium* sp.	α-proteobacteria	72	WT	C	20 - 45	4.5 - 5	8 - 10	1 - 4	0.1 - 2	0.1 - 0.5	S	S	S	R	R
**VAFP4**		Inceptisol	Black	Field	*Rhizobium* sp.	α-proteobacteria	48	WT	C	20 - 45	4.5 - 5	8 - 10	1 - 4	0.1 - 2	0.1 - 0.5	R	R	R	R	R
**VAFP8**		Inceptisol	Black	Field	*B. yuanmingense*	α-proteobacteria	72	WT	C	20 - 45	4.5 - 5	8 - 10	1 - 4	0.1 - 2	0.1 - 0.5	S	S	S	R	R
**VAFP9**		Inceptisol	Black	Pot	*B. elkanii*	α-proteobacteria	72	WT	C	20 - 45	4.5 - 5	8 - 10	1 - 4	0.1 - 2	0.1 - 0.5	S	S	S	R	R
**VAFW1**		Inceptisol	White	Field	*Rhizobium* sp.	α-proteobacteria	48	WT	C	20 - 45	4.5 - 5	8 - 10	1 - 4	0.1 - 2	0.1 - 0.5	R	R	R	R	R
**VAFW14**		Inceptisol	White	Field	*Rhizobium* sp.	α-proteobacteria	48	WT	C	20 - 45	4.5 - 5	8 - 10	1 - 4	0.1 - 2	0.1 - 0.5	S	S	S	R	R
**VAFW15**		Inceptisol	White	Pot	*Rhizobium* sp.	α-proteobacteria	72	WT	C	20 - 45	4.5 - 5	8 - 10	1 - 4	0.1 - 2	0.1 - 0.5	S	S	S	R	R
**VAFW5**		Inceptisol	White	Pot	*R. pusense*	α-proteobacteria	48	WT	C	20 - 45	5	8 - 10	1 - 4	0.1 - 2	-	S	S	S	R	S
**VAP1**	Aragua without Fertilizer	Inceptisol	Black	Pot	*R. phaseoli*	α-proteobacteria	72	W	C	20 - 45	4.5 - 5	8 - 10	1 - 4	0.1 - 2	0.1 - 1	R	S	S	R	R
**VAP4**		Inceptisol	Black	Pot	*R. tropici*	α-proteobacteria	96	WT	C	20 - 45	4.5 - 5	8 - 10	1 - 4	0.1 - 2	0.1 - 2	S	S	S	R	R
**VAP8A**		Inceptisol	Black	Pot	*Rhizobium* sp.	α-proteobacteria	120	WT	C	20 - 45	5	8 - 10	1 - 4	0.1 - 2	0.1	R	R	R	R	S
**VAP9**		Inceptisol	Black	Field	*R. phaseoli*	α-proteobacteria	48	WT	SS	20 - 45	4.5 - 5	8 - 10	1 - 4	0.1 - 2	0.1 - 1	R	S	S	R	S
**VAW10**		Inceptisol	White	Pot	*Rhizobium* sp.	α-proteobacteria	96	WT	SS	20 - 45	4.5 - 5	8 - 10	1 - 4	0.1 - 2	0.1 - 0.5	R	R	R	R	R
**VAW15**		Inceptisol	White	Pot	*Rhizobium* sp.	α-proteobacteria	48	T	SS	20 - 45	5	8 - 10	1 - 4	0.1 - 2	0.1	R	S	R	R	R
**VAW3**		Inceptisol	White	Field	*B. liaoningense*	α-proteobacteria	96	WT	C	20 - 45	4.5 - 5	8 - 10	1 - 4	0.1 - 2	0.1 - 0.5	R	S	R	R	R
**VAW5**		Inceptisol	White	Pot	*Rhizobium* sp.	α-proteobacteria	96	W	C	20 - 45	4.5 - 5	8 - 10	1 - 4	0.1 - 2	0.1 - 0.5	S	S	S	R	R
**VAW6**		Inceptisol	White	Field	*R. mesoamericanum*	α-proteobacteria	96	T	SS	20 - 45	4.5 - 5	8 - 10	1 - 4	0.1 - 2	0.1 - 0.5	R	R	R	R	R
**VApP1**	Apure	Inceptisol	Black	Pot	*Rhizobium* sp.	α-proteobacteria	48	WT	C	20 - 45	4.5 - 5	8 - 10	1 - 4	0.1 - 2	0.1 - 0.5	S	S	R	R	S
**VApP10**		Inceptisol	Black	Pot	*Rhizobium* sp.	α-proteobacteria	48	W	C	20 - 45	4.5 - 5	8 - 10	1 - 4	0.1 - 2	0.1 - 2	R	S	R	R	R
**VApP5**		Inceptisol	Black	Pot	*Rhizobium* sp.	α-proteobacteria	48	WT	C	20 - 45	4.5 - 5	8 - 10	1 - 4	0.1 - 2	0.1 - 0.5	R	S	R	R	R
**VApP8**		Inceptisol	Black	Pot	*Rhizobium* sp.	α-proteobacteria	48	W	C	20 - 45	4.5 - 5	8 - 10	1 - 4	0.1 - 2	0.1 - 2	R	S	R	R	R
**VAmP2A**	Amazonas	Oxisol	Black	Pot	*Rhizobium* sp.	α-proteobacteria	48	W	C	20 - 45	4.5 - 5	8 - 10	1 - 4	0.1 - 2	0.1 - 1	S	S	S	R	R
**VAmP8**		Oxisol	Black	Pot	*Burkholderia* sp.	β-proteobacteria	96	W	C	20 - 45	4.5 - 5	8 - 10	1 - 4	0.1 - 2	0.1 - 1	S	S	S	S	R
**VAmW2**		Oxisol	White	Pot	*Rhizobium* sp.	α-proteobacteria	96	W	C	20 - 45	4.5 - 5	8 - 10	1 - 4	0.1 - 2	0.1 - 0.5	S	S	S	R	R
**VMP1**		Ultisol	Black	Pot	*Rhizobium* sp.	α-proteobacteria	48	WT	C	20 - 45	4.5 - 5	8 - 10	1 - 4	0.1 - 2	0.1 - 0.5	R	S	R	R	R
**VMP18**		Ultisol	Black	Pot	*Rhizobium* sp.	α-proteobacteria	72	WT	C	20 - 45	4.5 - 5	8 - 10	1 - 4	0.1 - 2	0.1 - 0.5	R	S	R	R	R

**VMP2**		Ultisol	Black	Pot	*Rhizobium* sp.	α-proteobacteria	48	WT	C	20 - 45	4.5 - 5	8 - 10	1 - 4	0.1 - 2	0.1 - 0.5	S	R	S	R	R
**VMP23**		Ultisol	Black	Pot	*R. phaseoli*	α-proteobacteria	72	WT	C	20 - 45	4.5 - 5	8 - 10	1 - 4	0.1 - 2	0.1 - 2	S	S	S	R	R
**VMP3**	Mérida	Ultisol	Black	Pot	*Rhizobium* sp.	α-proteobacteria	72	WT	C	20 - 45	4.5 - 5	8 - 10	1 - 4	0.1 - 2	0.1 - 1	R	R	R	R	R
**VMP6**		Ultisol	Black	Pot	*Burkholderia* sp.	β-proteobacteria	72	WT	C	20 - 45	4.5 - 5	8 - 10	1 - 4	0.1 - 2	0.1 - 0.5	R	S	R	R	R
**VMP8**		Ultisol	Black	Pot	*R. pisi*	α-proteobacteria	72	WT	C	20 - 45	4.5 - 5	8 - 10	1 - 4	0.1 - 2	0.1 - 0.5	S	S	S	R	R
**VMW1**		Ultisol	White	Pot	*Rhizobium* sp.	α-proteobacteria	72	WT	C	20 - 45	4.5 - 5	8 - 10	1 - 4	0.1 - 2	0.1 - 0.5	S	S	S	R	R
**VMW4**		Ultisol	White	Pot	*Rhizobium* sp.	α-proteobacteria	72	WT	C	20 - 45	4.5 - 5	8 - 10	1 - 4	0.1 - 2	0.1 - 0.5	S	S	S	R	R
**VMW7**		Ultisol	White	Pot	*Rhizobium etli*	α-proteobacteria	72	WT	C	20 - 45	4.5 - 5	8 - 10	1 - 4	0.1 - 2	0.1 - 0.5	S	S	S	R	R
**VTrP29**		Ultisol	Black	Pot	*Rhizobium* sp.	α-proteobacteria	48	WT	C	20 - 45	5	8 - 9	1 - 4	0.1 - 2	0.1 - 0.5	S	S	R	R	S
**VTrP4**	Trujillo	Ultisol	Black	Pot	*Rhizobium* sp.	α-proteobacteria	144	T	C	20 - 45	-	8 - 9	1	0.1	-	S	S	S	S	S
**VTrW6**		Ultisol	White	Pot	*R. pusense*	α-proteobacteria	48	WT	C	20 - 45	4.5 - 5	8 - 10	1 - 4	0.1 - 2	0.1	S	S	S	R	S
**VGP2**		Vertisol	Black	Pot	*M. plurifarium*	α-proteobacteria	48	W	C	20 - 45	4.5 - 5	8 - 10	1 - 4	0.1 - 2	0.1	S	S	S	R	R
**VGP2B**		Vertisol	Black	Pot	*B. embrapense*	α-proteobacteria	72	WT	C	20 - 45	5	8 - 10	1 - 4	0.1 - 2	-	R	S	S	R	R
**VGP4**		Vertisol	Black	Field	*Rhizobium etli*	α-proteobacteria	96	W	C	20 - 45	4.5 - 5	8 - 10	1 - 4	0.1 - 2	0.1 - 1	R	S	S	R	R
**VGP6**		Vertisol	Black	Pot	*Bradyrhizobium* sp.	α-proteobacteria	96	W	C	20 - 45	4.5 - 5	8 - 10	1 - 4	0.1 - 2	0.1 - 0.5	R	S	R	R	R
**VGP9**	Guárico	Vertisol	Black	Pot	*Bradyrhizobium* sp.	α-proteobacteria	48	WT	C	20 - 45	4.5 - 5	8 - 10	1 - 4	0.1 - 2	0.1 - 0.5	R	S	R	R	R
**VGW15C**		Vertisol	White	Field	*R. pusense*	α-proteobacteria	96	WT	C	20 - 45	4.5 - 5	8 - 10	1 - 4	0.1 - 2	0.1 - 0.5	S	S	R	R	R
**VGW2**		Vertisol	White	Field	*M. plurifarium*	α-proteobacteria	48	W	C	20 - 45	4.5 - 5	8 - 10	1 - 4	0.1 - 2	0.1 - 2	S	S	S	R	R
**VGW5**		Vertisol	White	Pot	*Rhizobium* sp.	α-proteobacteria	96	WT	C	20 - 45	5	8 - 10	1 - 4	0.1 - 2	-	R	S	R	S	R
**VGW7B**		Vertisol	White	Field	*P. phymatum*	β-proteobacteria	120	WT	C	20 - 45	5	8 - 10	1 - 4	0.1 - 2	0.1	R	S	S	S	S
**VLaP2**		Vertisol	Black	Pot	*Rhizobium* sp.	α-proteobacteria	48	T	C	20 - 45	4.5 - 5	8 - 10	1 - 4	0.1 - 2	0.1 - 0.5	R	R	S	R	R
**VLaP5**		Vertisol	Black	Field	*R. pusense*	α-proteobacteria	72	WT	C	20 - 45	4.5 - 5	8 - 10	1 - 3	0.1 - 2	0.1 - 0.5	R	S	R	R	R
**VLaW1**	Lara	Vertisol	White	Pot	*Rhizobium* sp.	α-proteobacteria	96	W	C	20 - 45	4.5 - 5	8 - 10	1 - 4	0.1 - 2	-	R	S	S	S	S
**VLaW27**		Vertisol	White	Field	*B. yuanmingense*	α-proteobacteria	72	W	C	20 - 45	4.5 - 5	8 - 10	1 - 4	0.1 - 2	0.1 - 0.5	R	S	R	R	R
**VLaW3**		Vertisol	White	Pot	*B. embrapense*	α-proteobacteria	48	WT	C	20 - 45	4.5 - 5	8 - 10	1 - 4	0.1 - 2	0.1 - 1	R	R	R	R	R
**VLaW4**		Vertisol	White	Pot	*P. phymatum*	β-proteobacteria	96	W	C	20 - 45	5	8 - 10	1 - 4	0.1 - 2	-	R	S	S	S	R

All names included sampling sites and host e.g.: VTrW6, V (Venezuela) - Tr (Trujillo) – W (white cultivar); VGP2, V (Venezuela) - G (Guárico) – P (black cultivar),
In MLST, B.: *Bradyrhizobium* . R.: *Rhizobium*. P.: *Paraburkholderia*. M.: *Mesorhizobium*.
Phenotype: Growth rates are based on hours in YMA. Color: transparent (T), white–transparent (WT), and white (W). Texture: sticky (SS), and creamy (C).
R: resistant strains (the strains could grow as good or weak rate compared with the control). S: sensitive (strains did not grow).
